# Animated virtual characters to explore audio-visual speech in controlled and naturalistic environments

**DOI:** 10.1038/s41598-020-72375-y

**Published:** 2020-09-23

**Authors:** Raphaël Thézé, Mehdi Ali Gadiri, Louis Albert, Antoine Provost, Anne-Lise Giraud, Pierre Mégevand

**Affiliations:** 1 Department of Basic Neurosciences, University of Geneva, Campus Biotech, Chemin des Mines 9, 1202 Geneva, Switzerland; 2 Human Neuroscience Platform, Fondation Campus Biotech Geneva, Geneva, Switzerland; 3 Division of Neurology, Geneva University Hospitals, Geneva, Switzerland

**Keywords:** Language, Sensory processing

## Abstract

Natural speech is processed in the brain as a mixture of auditory and visual features. An example of the importance of visual speech is the McGurk effect and related perceptual illusions that result from mismatching auditory and visual syllables. Although the McGurk effect has widely been applied to the exploration of audio-visual speech processing, it relies on isolated syllables, which severely limits the conclusions that can be drawn from the paradigm. In addition, the extreme variability and the quality of the stimuli usually employed prevents comparability across studies. To overcome these limitations, we present an innovative methodology using 3D virtual characters with realistic lip movements synchronized on computer-synthesized speech. We used commercially accessible and affordable tools to facilitate reproducibility and comparability, and the set-up was validated on 24 participants performing a perception task. Within complete and meaningful French sentences, we paired a labiodental fricative viseme (i.e. /v/) with a bilabial occlusive phoneme (i.e. /b/). This audiovisual mismatch is known to induce the illusion of hearing /v/ in a proportion of trials. We tested the rate of the illusion while varying the magnitude of background noise and audiovisual lag. Overall, the effect was observed in 40% of trials. The proportion rose to about 50% with added background noise and up to 66% when controlling for phonetic features. Our results conclusively demonstrate that computer-generated speech stimuli are judicious, and that they can supplement natural speech with higher control over stimulus timing and content.

## Introduction

Natural communication is a multimodal experience. Auditory and visual features of speech are perceived as a single event despite the fact that sensory stimuli from each modality are initially processed independently. Understanding this integrative multi-sensory process has become an active topic of research for speech neuroscientists.

Studying natural speech requires robust ecological material. Unfortunately, compared to existing datasets for auditory speech, which are extensive, the corpus of audiovisual speech is poor^1^. Producing video recordings requires time and specialized material, and researchers generally resort to the use of low-quality cameras or rely on pre-existing film material^2^. This results in the multiplication of local datasets, generally in the local spoken language^3,4^, with different designs or features, which are not comparable to one another.

The McGurk effect^5^, and related illusory perceptions^6^ that result from incongruent visual and auditory speech tokens, have been extensively used as a proxy for studying multimodal speech perception. Despite great scientific acceptance, the literature reports enormous variability and, unsurprisingly, extrapolation to natural speech processing is unmet. Perception of the illusion varies between experiments, even within the same study^7^, but also between individuals and across stimuli^8,9^.

Many reasons can explain this large variability. To begin with, the original stimuli used by McGurk and MacDonald^5^ are no longer available, rendering proper replication impossible, and there is now a constellation of “McGurk stimuli” in use^10^. Since experimental stimuli are made in-lab and new stimuli are made for every experiment, this adds considerable variability. At best, published studies mention hiring actors^11–13^, but otherwise they simply refer to a “native speaker”^14,15^, suggesting the latter was rather recruited from colleagues and friends for convenience. Visual and acoustic features of speech are however extremely variable between individuals, and different speakers may induce more or less frequent illusory percepts across listeners^16,17^ with no metric to normalize intelligibility^10^. Incongruent stimuli are designed manually, swapping audio-visual tracks, but the voicing must be exactly identical in order to avoid a mismatch sensation, a difficult task for non-professionals. Although perfect dubbing is not required for inducing the illusory percept^18,19^, excessive cross-modal incongruency induces a mismatch sensation^20^, which can be detrimental to the illusion^21^.

A more standardized approach is therefore crucial, something speech synthesis and animated virtual characters can provide. It has long been known that a wide variety of stimuli can induce McGurk effects, including artificial computer-generated faces^22^, speakers with paralyzed facial muscles^23,24^, point-light speech^25^ or by occluding part of the face, leaving only the mouth^26^. This suggests that minimal cues are required for a human listener to perceive synthetic audio-visual speech as natural, yet applications to speech perception research remain anecdotal. Published initiatives for developing artificial audio-visual speech are nonetheless numerous. The *Synface* project^27,28^, a speech-driven system for telecommunication, developed a 3D animated face model following a set of deformation parameters to simulate articulation. The animations however focused on lip articulation with no facial micro-gestures, causing a feeling of uneasiness known as the “uncanny valley”, and the project was eventually abandoned with the advent of video calling. *Greta*^29^ was a prototype of a virtual agent able to communicate using speech and nonverbal gestures; however, to our knowledge, it has not been used to study speech processing. Schabus et al.^30,31^ also developed a corpus of speech data using facial motion capture and Hidden Markov Model-based speech synthesis. Other approaches implemented Facial Acting Coding Systems with lip synchronization^32–34^, anatomy-based models with MRI imaging^35^ or biomechanics^36^. Most of this work was published with relatively scant methodological details, and the stimuli are now unavailable. Furthermore, these previous attempts suffered from an overwhelming technical complexity relative to the needs of experimental design for speech neuroscientists, steering them toward cruder solutions like filming actors and doing basic editing, with the caveats that we mentioned above.

**Figure 1:**
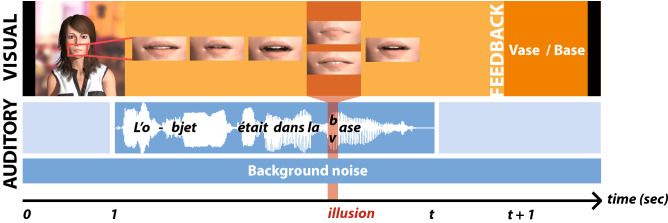
Task design. The figure illustrates the time course of one trial. The identity of the virtual character (visual features and voice) varied from trial to trial, as well as the sentence and type of condition. The virtual character first appeared on screen, remaining silent, but with an ongoing background noise. After one second, the character started speaking. In this example, the uttered sentence is *l’objet était dans la vase/base*, which translates to *the object was in the mud/base*. One second after the character stopped talking, two buttons popped on the bottom of the screen, proposing two possible answers to what was uttered (i.e. *vase* or *base*). The two options remained on screen until the participant selected one with the arrows from the keyboard. The screen then faded to black and a new trial started.

In the present study, we introduce a simple stimulus design alternative to study audiovisual speech with computer-generated speakers and synthetic speech. Our overarching goal was to provide full control over the contents and timing of speech stimuli and thus reduce variability across studies due to disparate stimuli. Our approach is based on commonly accessible tools from the Adobe Suite, a paid Unity asset and a basic commercial speech synthesizer. Our specific aim was to develop realistic yet controllable speech features in both modalities. To demonstrate the validity of our design, we asked participants to undergo a speech perception task where we voluntarily introduced one mismatched audiovisual speech token (see Fig. 1). We embedded these mismatched phoneme-viseme pairs within complete meaningful French sentences. As a consequence of the mismatching, one word would turn into another depending on which one of the auditory or the visual cue drove the participant’s perception at the single-trial level. Our stimuli were thus similar to those of a McGurk experiment, with the nuance that the particular auditory and visual cues that we selected caused perception to reflect either the auditory or the visual speech token. We chose this approach to illustrate how gaining full control over the timing and contents of audiovisual speech stimuli allows creating seamless illusions that would otherwise be very difficult to induce. Note that our methodology can be applied more broadly to any type of speech stimuli and in any language. We tested the occurrence of the illusion and the influence of varying two key physical parameters known to modulate its frequency, background noise^37,38^ and audio-visual lag^16,39^. Varying some parameters while controlling others, such as spatial frequency or intonation, is a key feature for a balanced experimental design. The result is a simple, elegant and easy-to-use naturalistic audio-visual speech paradigm with an increased level of control over all the parameters.

## Results

### Analysis of the perceptual illusion

We designed a set of sentences written in French and uttered by a virtual character (see Fig. 1). The sentences were built in pairs (see Table 1), identical except for one target phoneme that could be either a labio-dental fricative (i.e. /v/ or /f/) or a bi-labial plosive (i.e. /b/ or /p/). These sentences were either congruent, with identical auditory and visual speech cues, or incongruent, where the auditory cue of the target phoneme was matched with the viseme corresponding to the alternative target phoneme. Importantly, all sentences remained semantically and syntactically correct. In total there were two factors (Congruence and Articulation) with two levels each (the viseme-phoneme pair is Congruent or Incongruent; the articulated *viseme* is Fricative or Plosive), with the highest rate of illusory perception expected to occur in an Incongruent sentence with a fricative visual cue (and thus a Plosive auditory cue). Perception was defined as correct if it corresponded to the auditory cue.

**Table 1:** List of sentences employed in the task and presented in pairs of phoneme fricative vs. plosive (in French).

Fricative	Plosive
L'objet était dans la vase	L'objet était dans la base
Ils étaient prisonniers de la vase	Ils étaient prisonniers de la base
Je ne sais pas, je ne l'ai pas vu	Je ne sais pas, je ne l'ai pas bu
Pendant le match je n'ai rien vu	Pendant le match je n'ai rien bu
Tu viens voir ?	Tu viens boire ?
Il n'y a rien à voir	Il n'y a rien à boire
C'est une vache blanche	C'est une bâche blanche
Regarde sous la vache	Regarde sous la bâche
Elle est fatiguée de verser	Elle est fatiguée de bercer
Elle chantait pendant qu'elle versait	Elle chantait pendant qu'elle berçait
Au zoo j'ai vu un faon	Au zoo j'ai vu un paon
Dans la forêt j'ai croisé un faon	Dans la forêt j'ai croisé un paon
J'ai pris ton fou	J'ai pris ton pouls
Il saute comme un fou	Il saute comme un pou
Elle a des jambes effilées	Elle a des jambes épilées
Il est blond avec une figure longue et effilée	Il est blond avec une figure longue et épilée
Il faut retirer les fils	Il faut retirer les piles
J'ai branché les fils électriques	J'ai branché les piles électriques

There were four combinations of Congruence and Articulation for each of the 18 sentences in our stimulus set. Each one of these sentences was uttered by 6 individual virtual characters, for a total of 432 individual audiovisual sentences. Because of the large number of stimuli, each one of 24 participants were presented one half of the stimuli, the selected stimuli being counterbalanced across participants. We analyzed the trials with no audiovisual lag and with a background noise level similar to the voice level. The participants reported a correct perception in 60.6 ± 15.7% of the incongruent trials with a fricative visual cue (about 40% of perceptions were thus illusory), which is the condition with the lowest rate of correct perception.

We found a main effect of Congruence (F_(1,23)_ = 47.17, p < 0.001, η^2^_p_ = 0.672), that is congruent trials were correctly perceived more often than incongruent trials (Congruent: 82.6 ± 12%; Incongruent: 65 ± 15%). The mean difference between congruent and incongruent trials was − 17.6% [95.0% CI − 23, − 13]. The p-value of the two-sided permutation t-test was < 0.001.

We also found a main effect of Articulation (F_(1,23)_ = 4.89, p = 0.037, η^2^_p_ = 0.175), meaning that trials with a plosive viseme were correctly perceived (77.4 ± 14.1%) more often than trials with a fricative viseme (70.4 ± 17.7%). The mean difference between trials with plosive and fricative visemes was − 6.94% [95.0% CI − 12, − 1.6]. The p-value of the two-sided permutation t-test was 0.0144.

Overall, the rate of perceived illusion increased in trials with an incongruent fricative viseme and plosive phoneme, although there was no interaction (F_(1,23)_ = 0.618, p = 0.44, η^2^_p_ = 0.026).

Semantic expectations could make one of the words in the pair more likely to be perceived than the other, depending on the sentence’s meaning. To ensure that context did not significantly influence the rate of illusory perception, we asked a different group of French speakers (n = 220) to rate on a numerical scale the relative plausibility of each member of the sentence pairs in our stimulus set. Overall, four sentences (pairs 8, 10, 16 and 18 referring to Table 1) were considered unbalanced, with one member of the pair deemed much more plausible than the other. There was no correlation between the relative plausibility of sentence pairs and their tendency to induce illusory perception on incongruent trials with a fricative visual cue (Pearson’s correlation: r^2^ = 0.026; p = 0.91). We also reran the repeated-measures ANOVA without the 4 unbalanced sentences, and found essentially identical results: significant main effects of Congruence (F_(1,23)_ = 25.5; p < 0.001) and Articulation (F_(1,23)_ = 4.76, p = 0.04), with no interaction.

Further, we tested if any virtual character would influence the illusion rate, but there were no effects of gender (F_(1,7)_ = 2.07, p = 0.19) or of face (F_(2,14)_ = 1.02, p = 0.38).

**Figure 2:**
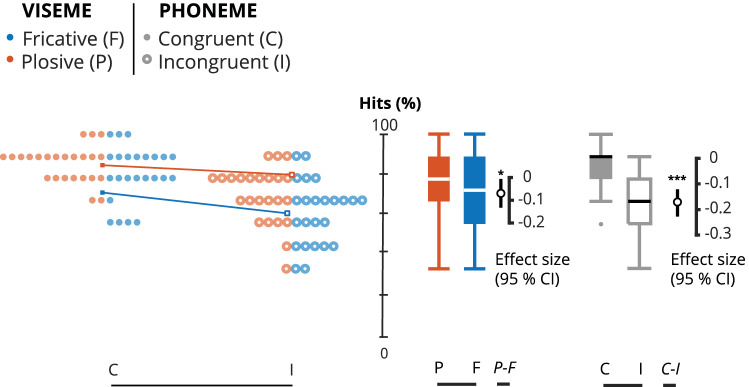
Estimation statistics for all stimuli. This modified Gardner-Altman estimation plot illustrates the hits, i.e. the percentage of trials for which what was “heard” corresponds to the auditory input, in all conditions. The left panel illustrates all observable values (hits %) for individual participants (averaged over sentences) plotted against the central axis. Viseme articulation (FRICATIVE or PLOSIVE) is identified by color and congruence (CONGRUENT or INCONGRUENT) is identified by shape, i.e. circle or dot. The mean of each group is illustrated with a squared data point of the corresponding color and shape. The effect size for each group comparison is illustrated on the right panel. The mean difference is illustrated with a black circle and the 95% confidence interval is illustrated with black vertical bars. For better readability and to illustrate the variability, the observable data is plotted again but as box-plot with the corresponding colors and shapes. The effect size is plotted against a floating axis on the right but the mean difference is aligned on the mean of the comparison group.

All effects are reported in Fig. 2.

### Modulating parameters

The lag between visual and acoustic cues as well as background noise are known to influence the occurrence of a McGurk illusion. We therefore generated stimuli with 3 levels of background noise for a voice level held constant (BACKGROUND: 50%, 75% or 100%; voices are set at 75%) and 3 levels of audiovisual asynchrony (SHIFT: − 100 ms, 0 ms and + 100 ms; a negative value means that the audio is shifted to occur before the visual). The audiovisual shift was applied to the entire sentence. We tested whether varying the stimulus physical features could modulate the illusory percept rate. In order to keep a balanced design, the participants were subjected to all combinations of Congruence and Articulation in all 9 combinations of physical features. However, only incongruent trials with a fricative viseme and plosive phoneme are of interest, and thus analyzed here.

We found a main effect of shift (F_(1,23)_ = 10.81, p = 0.001, η^2^_p_ = 0.497) and a main effect of background noise (F_(1,23)_ = 19.32, p < 0.001, η^2^_p_ = 0.637). There was no effect of interaction between the two.

Subjects displayed significantly poorer accuracy—increased illusory percept rate—on trials with a higher background noise (100%) relative to speech (75%) as opposed to trials with similar or lower background noise (75%, mean Δ = − 7.6 ± 1.7%, p = 0.001; 50%, mean Δ = − 10.3 ± 2.1%, p < 0.001; Bonferroni corrected). Subjects were significantly less accurate when there was no shift rather than a − 100 ms shift (mean Δ = − 8.3 ± 1.8%, p = 0.001; Bonferroni corrected). The absence of shift also induced more mistakes than a shift at + 100 ms, although it did not differ significantly (mean Δ = − 4.9 ± 2.4%, p = 0.15; Bonferroni corrected).

Therefore, the occurrence of the perceptual illusion (46.3 ± 17.5%) was higher in the absence of any audio-visual shift between the audio and the visual track, and with louder background noise.

**Figure 3:**
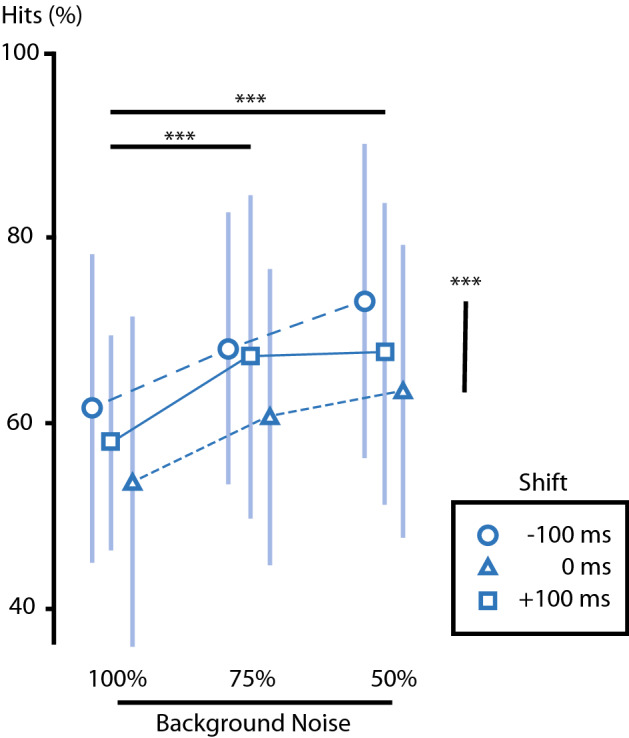
Modulating parameters. The plot illustrates the hits (%) of the condition FRICATIVE-INCONGRUENT, i.e. the percentage of trials for which the participants’ perception corresponded to the auditory input. Trials with different level of background noise are gathered as columns as indicated at the bottom of the plot. Trials with different audio-visual shifts are identified with different shapes as indicated in the legend. The shape also illustrates the mean value across participants for a given condition. The standard deviation for each condition of background noise and shift is illustrated with vertical bars. Paired differences between levels of background noise are illustrated with horizontal black bars and paired differences between level shifts are illustrated with vertical black bars. Significance is indicated with asterisks.

All effects are reported in Fig. 3.

### Sentence-wise comparison

We looked at individual stimuli to test if there was a variability between different sentences. For this analysis, only trials with optimal parameters (i.e. no audiovisual lag and a background noise level higher than the auditory input level, cf. Fig. 3) were considered.

Hits between incongruent trials with a /b/ phoneme and /v/ viseme and incongruent trials with a /p/ phoneme and a /f/ viseme differed significantly (t_(16)_ = − 5.71, p < 0.001). That is, the participants reported a correct percept with the /f-p/ trials (mean 79.2 ± 15%) at a higher rate than with the /b-v/ trials (mean 33.4 ± 18%). The mean difference was − 45.8% [95.0% CI − 29.8, − 59]. The p-value of the two-sided permutation t-test was < 0.001.

This is also true for congruent trials with /b/ phoneme-viseme pairs (69.2 ± 16.9%) as compared to trials with /p/ phoneme-viseme pair (95.4 ± 1.6%) which differed significantly (t_(16)_ = 4.35, p < 0.001). The mean difference was − 26.2% [95.0% CI − 18.2, − 38.3]. The p-value of the two-sided permutation t-test was < 0.001.

**Figure 4:**
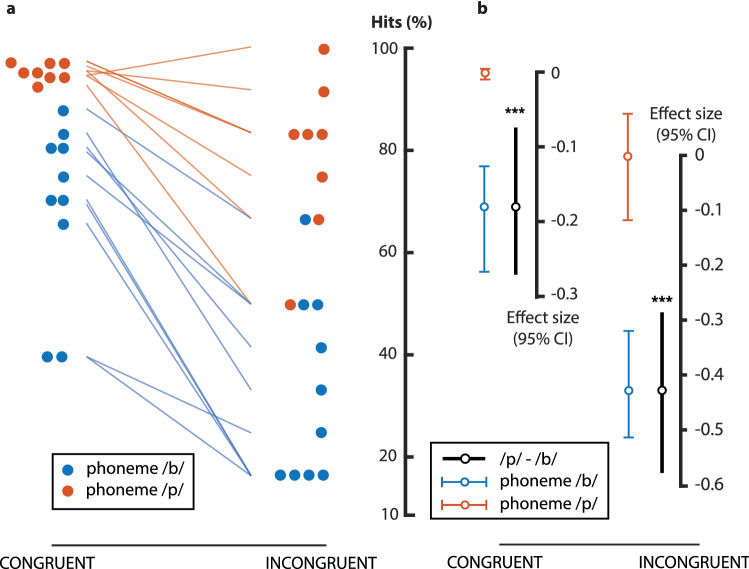
Sentence-wise comparison. This modified Gardner-Altman estimation plot illustrates the hits, i.e. the percentage of trials for which what was “heard” corresponds to the auditory input, for the trials with no lag between the auditory and visual inputs and with a background noise at a higher level than the auditory input. The congruent trials are on the left and the incongruent trials on the right. The sentences with a /b/ phoneme are displayed in blue while sentences with a /p/ phoneme are displayed in orange. (**A**) displays all observable values (% hits) for individual sentences (averaged over participants) plotted against the central axis. The difference between congruent and incongruent trials for a given sentence is illustrated with a color-coded slopegraph. (**B**) Displays the mean difference between /b/ and /p/ phoneme for congruent and incongruent trials. The effect size is plotted on a floating axis on the right. The mean difference is illustrated with a black circle and the 95% confidence interval is illustrated with black vertical bars. To ease visualization, hits are reported again in (**B**) with the mean value illustrated with a circle colored as the corresponding phoneme category and the standard deviation illustrated with corresponding colored vertical bars. The mean difference is aligned on the median of the comparison group. Overall, we observe a higher rate of hits for sentences with a /p/ phoneme.

All effects are reported in Fig. 4.

### Analysis of the illusion rate on /b-v/ sentences

Following up on the above results, Congruence and Articulation was compared again, this time only considering the trials comprising sentences with voiced phoneme and viseme (/b/ and /v/) and including the optimal parameters of background noise (100%) and audio-visual shift (0 ms).

We found a main effect of Congruence (F_(1,23)_ = 30.26, p < 0.001, η^2^_p_ = 0.568), that is congruent trials were correctly perceived more often than incongruent trials (Congruent: 72.9 ± 23.9%; Incongruent 47.9 ± 27.9%). The mean difference between congruent and incongruent trials was − 25% [95.0% CI − 34.2 − 17.5]. The p-value of the two-sided permutation t-test was < 0.001.

There was a main effect of Articulation, (F_(1,23)_ = 10.33, p = 0.004, η^2^_p_ = 0.31), that is trials with a plosive viseme (70.8 ± 22.2%) were correctly perceived more often than trials with a fricative viseme (50 ± 30.9%). The mean difference between trials with plosive and fricative visemes was − 20.8% [95.0% CI − 31.2, − 10.4]. The p-value of the two-sided permutation t-test was < 0.001.

Importantly, we observed an effect of interaction (F_(1,23)_ = 4.83, p = 0.038, η^2^_p_ = 0.174) between congruence and articulation. Overall, the participants reported perceptual illusions for the incongruent trials with a fricative viseme and plosive phoneme in about two thirds of the trials (number of hits was 33.3 ± 27.4%).

To ensure that these findings were not due to unbalanced semantic expectations, we also ran the repeated-measures ANOVA without the sentence pairs deemed unbalanced. We again found main effects of Congruence (F_(1,23)_ = 22.05; p < 0.001) and Articulation (F_(1,23)_ = 9.37; p = 0.006) as well as a significant interaction (F_(1,23)_ = 4.5; p = 0.045). These results indicate that semantic expectations did not play an outsized role in the rate of illusory perception.

**Figure 5:**
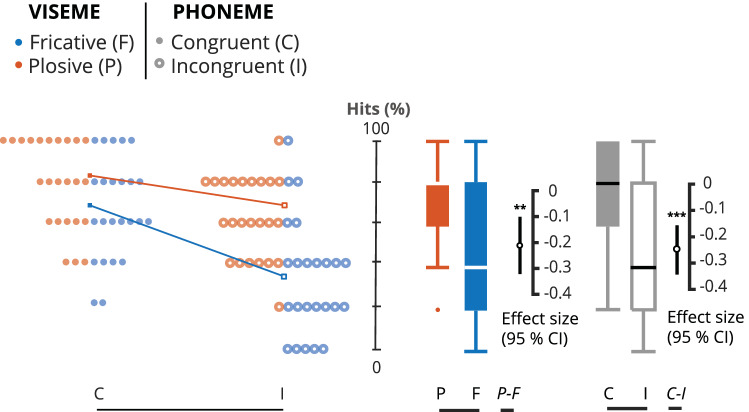
Final estimation statistics for voiced stimuli. This modified Gardner-Altman estimation plot illustrates the hits in all conditions but only for the trials comprising sentences with the phoneme and viseme /b/ and /v/. The left panel illustrates all observable values (hits %) for individual participants (averaged over sentences) plotted against the central axis. Viseme articulation (FRICATIVE or PLOSIVE) is identified by color and congruence (CONGRUENT or INCONGRUENT) is identified by shape, i.e. circle or dot. The mean of each group is illustrated with a squared data point of the corresponding color and shape. The effect size for each group comparison is illustrated on the right panel. The mean difference is illustrated with a black circle and the 95% confidence interval is illustrated with black vertical bars. For better readability and to illustrate the variability, the observable data is plotted again but as box-plot with the corresponding colors and shapes. The effect size is plotted against a floating axis on the right but the mean difference is aligned on the median of the comparison group.

All effects are reported in Fig. 5.

## Discussion

Traditionally, audiovisual speech stimuli, like the mismatched stimuli that induce the McGurk effect, are designed from homemade video recordings, yielding a low level of control and reproducibility. Creating synthetic audio-visual stimuli is not a new idea^40^. However, in the past, it has involved modelling a virtual 3D articulating face from scratch^41,42^ or video morphing techniques to rewrite segments from real recorded speakers^43–45^. Those methods have found little applicability for the speech scientific community in need to develop new paradigms, and we therefore set out to develop a simpler, affordable and more versatile solution. We used a common commercial software to design animated virtual characters and synthesized speech, yielding a naturalistic presentation of audiovisual speech. We demonstrate that these stimuli are appropriate for multisensory speech perception research by successfully using them to generate a perceptual illusion.

Humans are quite good at perceiving degraded speech^46,47^; thus, the aim should not be to develop absolutely perfect audiovisual speech stimuli if the cost and labor are unreasonably high. Here, we contend that being able to parametrize the stimuli at will (e.g. by selecting and mismatching auditory and visual cues) while maintaining complete control over stimulus timing primes over perfect photorealism. Indeed, we demonstrate that it is sufficient to associate the articulation of a number of key visemes to a greater number of phonemes and to interpolate the resulting keyframes for co-articulation. Particular attention was given to provide the virtual characters with facial micro-gestures, eye saccades and swaying, which gives the character a feeling of liveliness and increases the comfort of the participants toward the virtual characters. While the feeling of immersion is generally overlooked, it is known to induce more realistic behavior from the participants^48,49^.

We preferred a variant to the classical McGurk fusion illusion, where a labio-dental fricative viseme (i.e. /v/ or /f/) was paired with a bi-labial plosive phoneme (i.e. /b/ or /p/). With this design there is no fused perception: the listener perceives either /b/ or /v/, which allowed us to embed the mismatched syllables in complete, meaningful sentences regardless of perception. To our knowledge, the study by Sams et al.^50^ is the only known attempt to use sentence-long verbal stimuli. However, their stimuli (3-word Finnish sentences) could induce the perception of non-words, thus creating semantically or syntactically incorrect sentences. Here, all perceptions induced by our stimuli were real words semantically and syntactically appropriate within the sentences. This is critical because meaningless syllables or non-words cannot be considered to be naturalistic speech; as a consequence, it is unclear whether the effects described with such stimuli truly reflect the processing of audiovisual speech rather than the resolving of a perceptual, syntactic or semantic conflict^10^.

The participants reported an illusory percept in more than half of the trials combining a /v/ viseme with a /b/ phoneme, which is within the range reported in the literature. The inter-participant variability was also similar to that of previous reports^9^. Surprisingly, there was a significant difference in the occurrence of the illusion between /b-v/ and /p-f/ phoneme-viseme pairs, the latter inducing the illusion in only a small proportion of trials. Although it was not investigated here, a possible explanation may rise from the phoneme linguistic features. The position of articulators distinguishes /p/ and /b/ from /f/ and /v/, where they are brought together for the latter and air is forced out. This results in a friction noise, hence the fricative denomination. This visual difference is what possibly induces the perceptual illusion. What differentiates between the /b-v/ and /p-f/ pairs is the voicing. The vocal chords do not vibrate with /p-f/ while they do for /b-v/, meaning they are aperiodic. Upon coarticulation with a vowel, the voiced transition of the second formant may reduce the difference of affrication between /b/ and /v/, which facilitates the illusion, and that may not be the case for /p/ and /f/ because the formant transition of /p/ occurs before vocalization, during aspiration^51^.

Raising the background noise above speech level significantly augmented the proportion of illusory percepts. By all accounts, visual and auditory processing is modulated by attention and various factors may drive perception toward a perceptual stream or another^52–54^. When the environment is noisy, speech perception relies more on visual cues to complement the less reliable auditory information^55,56^. In the case of mismatched audiovisual stimuli, relying more on visual cues may increase the likelihood of a false percept. Lag between modalities is also a known factor modulating multisensory integration. For very brief nonverbal stimuli, asynchrony can be detected with a sensitivity as high as 20 ms^57,58^. In the case of McGurk and similar speech stimuli, delaying the auditory stream relative to the visual cue may favor the influence of that modality and increase the occurrence of the illusion, with maximal frequency reported at lags up to 130–180 ms^16,39^. For this reason, we tested the rate of illusion with audiovisual asynchronies of − 100 and + 100 ms. Against expectations, we observed that an audiovisual lag did not increase the occurrence of the illusory percept. It has been shown that the time window of multisensory integration is less variable when longer stimuli are used^59^, and that using long and meaningful speech tokens rather than short or unintelligible fragments narrows this time period^60^. Thus, our long and correct sentences might have caused a narrowing of the time window of multisensory integration, compared to the meaningless utterances used in previous studies. This would have to be tested in future work but, nonetheless, it supports the idea that speech perception recruits a multimodal integrative network that is not perfectly reflected in nonverbal tasks^18^. Our results stress the importance of using well-controlled, naturalistic stimuli to explore multisensory effects in speech processing.

The high variability of illusory perception across participants and stimuli suggests that the grouped mean response frequency is an unsuitable measure of audio-visual speech integration and that each participant and trial should be considered individually. Our observations also call for caution when using a paradigm with videotaped actors to study audiovisual speech. Verbal stimuli with precise control over timing and acoustic and visual features are more desirable, which is feasible using virtual characters and synthesized speech. Recent advances in artificial intelligence are also promising for the future development of audio-visual speech animations^61–63^, although they generally require large input datasets to train a model. For instance, Suwajanakorn et al.^64^ were able to generate a long, non-existing audiovisual speech based on footage from a large databank of different public talks by the same speaker. Aneja & Li^65^ also developed coherent, live lip-synchronization of an avatar, a simple face cartoon, based on an audio stream. Those studies are so far only compatible with 2D flat videos, while the proposed method here is adaptable to virtual reality environments. Overall, we provide a simple, yet novel, approach for speech neuroscientists to adopt more standard and controlled designs in their work. Future developments may include a combination of virtual characters such as ours with neural network training for complete autonomy and increased flexibility. Because visual and auditory speech are both crucial for natural communication, with strong implication in various areas ranging from clinical rehabilitation to digital communication, it is essential to address the mechanisms of speech perception with robust and naturalistic material that is openly accessible to researchers.

## Materials and methods

### Participants

Twenty-four healthy participants with no hearing impairments and normal or corrected-to-normal vision (13 female, age ranging 22 to 44 y.o., mean 27.9 y.o.) were recruited to participate in this study. All participants provided informed consent in writing. They were either native French speakers or completely fluent in French. All procedures were approved by the local Ethics Committee (Commission cantonale d’éthique de la recherche sur l’être humain de la République et canton de Genève; project #2018-00911) and was conducted in accordance with the relevant Swiss laws and regulations and international guidelines (Declaration of Helsinki) on research on human subjects.

### Speech stimuli

We designed a set of computerized speech stimuli to manipulate the interaction between the visual and auditory modalities. The aim was to develop realistic yet controllable speech features in both modalities.

#### Auditory input

We composed 18 pairs of sentences in French (see Table 1). Sentences within each pair were identical except for one target word which differed by one consonant. A pair of target words always involved a bi-labial plosive consonant swapped with a labio-dental fricative consonant. More precisely, target words either paired /v/ with /b/ (n = 10) or /f/ with /p/ (n = 8). All sentences remained semantically and grammatically correct in every declination of a target word.

We used the software ReadSpeaker (www.readspeaker.com) to process all 36 written sentences into naturalistic auditory speech saved in Waveform Audio File Format (WAV). All stimuli were generated in two versions with a female and a male voice. The audio files were then manipulated with the software Audacity (www.audacity.fr) to obtain a lower pitch (− 10%) and higher pitch (+ 10%) version for each gender resulting in six distinct voices (3 female and 3 male) for all 36 sentences, therefore making 216 different stimuli.

#### Visual input

We designed six 3D computer-generated characters (3 females, 3 males; see supplementary Fig. 1) with the software *Adobe Fuse CC* (see supplementary Fig. 2A). The software has a large library of customizable bodies (weight, height, skin color, eyes color, hair… etc.), clothes and accessories. Each virtual character is then sent to *Adobe Mixamo* (see supplementary Fig. 2B), which provides a library of body animations for the character (here, a basic idle animation). Most importantly, it provides facial blendshapes, which are linear deformations of a 3D shape that can be dynamically modified with a slider to provide, for instance, articulatory movements of the mouth. Other examples of facial blendshapes are eye blinks, eyebrow raising, etc.

The complete virtual characters were exported and implemented in Unity 3D. A custom C# script randomly changed the value of facial blendshapes to add micro-movements to the face, but also eye blinks and eye gaze. The characters were programmed to look at a virtual camera (e.g. the screen) and to occasionally shift gaze between two close points to simulate eye saccades. These features were implemented to give the feeling of life to the virtual character.

#### Audiovisual stimuli

We built the audiovisual stimuli by associating each voice version of the 36 auditory inputs to a unique visual character (see supplementary Fig. 2C).

The *LipSync Pro* plugin was used to design the combinations of blendshapes used to generate articulatory mouth movements for specific visemes (e.g. AI, E, U, O, CDGKNRSTHYZ, FV, L, MBP, WQ or Rest). These directives could then be associated to an audio track in the form of key frames to create a sequence of lips movements matching the acoustic sequence (e.g. a sentence). Intensity of every keyframe was set to 70% except for the McGurk inducing visemes (B/V or P/F), which were set to 100%. This way, these visemes were slightly exaggerated so as to increase the potential of inducing an illusion, while the difference with other visemes was too small to be consciously perceived by the participants. For each sentence, several of these combinations of blendshapes are placed by hand on the audio track to create a sequence of facial and lips positions matching the sentence. This sequence is saved for each sentence. When the animation is playing, positions in the corresponding sequence are non-linearly interpolated along time to give a more realistic facial animation impression.

Please note that the visual appearance of the virtual characters, as well as the voices obtained by speech synthesis, are not based on real persons, and therefore cannot be used to identify recognizable persons.

### Experimental conditions

In this study we were interested in inducing a McGurk-like illusion to manipulate the audio-visual processing of speech. For this purpose, we defined four conditions to test the occurrence of the effect (congruence and articulation). These conditions were combined with nine additional conditions to test the optimal parameters inducing the illusion (lag and background noise).

For each pair of audiovisual stimuli, we swapped the keyframes corresponding to the differing phoneme in a target word. Because of the accuracy of computer-generated input, the sentences remained identical except for that phoneme whose visual and auditory features were mismatched. In other words, while the virtual character’s lips are articulating the sentence *l’objet était dans la base*, the auditory track is playing *l’objet était dans la vase* with identical alignment and rhythmicity. In total, there were 432 different stimuli. Half of them belonged to either condition CONGRUENT or INCONGRUENT, based on the phoneme correspondence to the viseme. Each group was further divided into conditions FRICATIVE (i.e. /f/ or /v/) and PLOSIVE (i.e. /p/ or /b/), based on the target words visually articulated (i.e. viseme). These resulted in 4 combinations for each of the 18 sentence pairs, each one being uttered by 6 distinct virtual characters. The illusory perception is expected to occur most often in the INCONGRUENT-FRICATIVE condition^6^ when a participant reports hearing a target word with a fricative phoneme, whereas the auditory input was a target word with a plosive phoneme.

Stimuli further belonged to three SHIFT conditions, with an offset of − 100, 0 or + 100 ms of the auditory input relative to the visual input (i.e. negative lag puts the audio before the video), and three BACKGROUND conditions, with a background noise level altered to 50%, 75% and 100% relative to a constant speech level at 75%. The background noise was an unintelligible recording of a café’s ambiance. These parameters were applied online on the audiovisual stimuli upon presentation. The illusion was expected to have a higher occurrence in one combination of SHIFT and BACKGROUND for trials INCONGRUENT-FRICATIVE.

Because of the exceedingly high number of stimuli across all conditions, any given participant watched only a subset of stimuli corresponding to half of the sentence pairs (n = 9) in all conditions (n = 36) distributed between two different characters uttering the stimuli. Any subset would always include five sentences with the consonant pair /b-v/ and four sentences with the consonant pair /p-f/. In the end, each participant was presented with 324 stimuli and, overall, each character was presented to four different participants, ensuring a balanced design. A Matlab script (The MathWorks, Natick. R2017a) built a stimulus sequence assigned to each participant, controlled for characters and stimuli repetition and randomized the trials. The script then exported the generated sequence in a CSV file later used for stimulus presentation with Unity.

### Task flow

For the experimental task, a GUI was designed with a collection of C# scripts in Unity 3D (Unity Technologies, www.unity.com). The script called the relevant files for audiovisual presentation. Facial animations were generated and executed on a fixed time basis set to the fixed rendering speed (60 FPS). The sequence of presentation and the parameters were additionally applied (i.e. audiovisual shift and/or level of background noise) as defined with a configuration document on a separate normalized .csv file.

The exact flow of a trial is detailed in Fig. 1 and in an example video (see supplementary video 1). At first, the virtual character was present on screen for 1 s and remained silent, although swaying and blinking, with background noise playing. The virtual character would then utter a sentence, as determined from the configuration file. Following the utterance the character remained silently on screen. After 1 s, two buttons popped up below the virtual character, who remained on screen. The buttons displayed the target word and its alternative (i.e. *vase* and *base*). Which button a word would appear on was determined at random. Participants responded by pressing the left or right arrow on the keyboard, corresponding to the left or right button. The scene on screen then faded to black for 1 s before starting a new trial. All answers and events were logged in an output Excel file.

Participants were instructed to report what they heard during the ongoing trial, focusing on the auditory percept in case of conflict. Classical McGurk studies with fusion stimuli have stressed the potential bias of referring to a specific modality^9^. In this study, however, we employed bistable stimuli meant to induce perception of either modality, rather than a fused percept. By asking participants to focus on their auditory perception, we reinforce the claim that a reported illusion results from processing of the visual cue. In fact, the role of selective attention for this kind of stimuli was shown to be exceedingly small: perception was not meaningfully driven by selective attention to the visual or the auditory component of the stimulus^66^.

### Material

The participants sat in a room with the experimenter and were allowed a break midway through the experiment. The experiment ran on a laptop computer Dell Precision 5,530 equipped with an Intel Core i7 8850H processor, a Nvidia quadro P1000 graphic card, 16GB Random-Access Memory and a 1,920 × 1,080 LED screen. The running operating system was Windows 10. All sounds were played on the computer’s native sound card and playback device at a sample rate set to 44,100 Hz which corresponds to the application audio sample rate. Unity audio sample rate was set to 44,100 Hz which corresponds to the sampling rate of the WAV audio file. Audio DSP Buffer size was set to 512 samples (which gives the best audio latency results on our setup without audio glitches) and filled online with the audio file. In this set-up, we obtained a maximum variability of ± 25 ms between the displayed visual facial animation and the auditory signal. This variability was stable over time. Graphics were displayed with a constant 60 FPS display rate during the experiment at full screen display. The screen had a diagonal length of 39.6 cm and the participants were seated at about 50 cm from the screen.

### Behavioral analysis

All output CSV files were imported in Matlab for data processing and the trials were sorted out by conditions. An answer was deemed correct for a given trial when the target word indicated as heard by the participant matched the target word of the auditory input. Any other answer was rated as incorrect. The percentage of correct responses (% Hits) between conditions was compared using repeated measures ANOVA (rmANOVA). Main effects were investigated with pairwise comparisons, Bonferroni corrected. Effect sizes are reported as partial eta square (η^2^_p_), which is the proportion of the effect with the error variance attributable to this effect. All statistical analyses were performed with the software *IBM SPSS Statistics* (IBM Corp. Released 2015 for Windows, Version 23.0.).

Because the meaning of a sentence could influence which of two words is expected to occur, we asked 220 French speakers to fill an online questionnaire and rate the relative plausibility of the members of our sentence pairs. Participants clicked on a link to a landing page with the instructions to the questionnaire and an example sentence not included in the study. The main questionnaire page showed all sentence pairs, each of which was immediately followed by a numerical scale ranging from 1 to 5. Sentences with a fricative syllable were on the left and sentences with a plosive syllable were on the right. The order of sentences was randomized across participants. Participants were instructed to rate the sentence pairs as follows: if they selected 1, they meant that the sentence on the left was considered much more plausible than the sentence on the right. Selecting 5 meant the opposite. Selecting 3 meant that both sentences were deemed equally plausible. For any sentence pair, we considered that scores below 2 or above 4 (averaged across participants) meant that the pair was unbalanced, with one sentence deemed more plausible. We also correlated the rate of illusion for incongruent trials with a fricative viseme and plosive phoneme sentence-wise to the average ratings of plausibility per sentence.

Figure illustrations were generated in Matlab and are inspired from the DABEST (‘data analysis with bootstrap-coupled estimation’) open-source toolbox^67^. The data are represented as Gardner-Altman estimation plots, which include all data points as a swarm plot and effect size as a bootstrap 95% confidence interval (95% CI). The effect sizes are an adjuvant to the statistical outcomes from null-hypothesis testing to improve the evaluation of significance^68^. The sampling-error curve was calculated with a randomization-based approach^67^. Using a bootstrap is more robust and versatile compared to parametric methods, particularly with non-normal distributions^69^. The effect sizes and CIs are reported as *effect size [CI width. lower bound; upper bound]*. 5,000 bootstrap samples were taken; the confidence interval is bias-corrected and accelerated. The p-values reported are the likelihoods of observing the effect sizes, if the null hypothesis of zero difference is true. For each permutation p-value, 5,000 reshuffles of conditions tested were performed. For aesthetic purposes, the figures were then adapted with the Adobe Illustrator software.

The analysis assessing the presence of the perceptual illusion tested the proportion of hits between conditions with a 2 × 2 rmANOVA and using Congruence (CONGRUENT, INCONGRUENT) and Viseme (FRICATIVE, PLOSIVE) as within-subject factors.

The analysis evaluating the effect of shift and background noise on the occurrence of the McGurk illusion tested the proportion of hits in the incongruent trials with a plosive phoneme and fricative viseme with a 3 × 3 rmANOVA using SHIFT (− 100 ms, 0 ms, + 100 ms) and BACKGROUND (50, 75, 100) as within-subject factors. Further, a 2 × 3 rmANOVA with gender (MALE, FEMALE) and face (1,2,3) as within-subject factors for trials with a shift at 0 ms and background noise at 75% was performed to test for an eventual influence of a virtual character on the illusion rates.

Additionally, the proportion of hits between trials with a /p/ phoneme and a /b/ were evaluated for congruent and incongruent trials averaged over participants with the parameters of shift and background noise that were identified as optimal in the previous analysis. The percentage of hits for the trials with the phoneme /b/ and the phoneme /p/ were compared using unpaired Student t-test.

## Supplementary information

Supplementary Information.

Supplementary Movie 1.

Supplementary Figure 1.

Supplementary Figure 2.

## Data Availability

The raw data generated by the behavioral experiment is available on the University of Geneva’s institutional repository, Yareta: https://doi.org/10.26037/yareta:shp4bepp7ngv3etn5u4xkms45q.
