# Supplementary material for: Animated virtual characters to explore audio-visual speech in controlled and naturalistic environments
Source: Sci Rep. 2020 Sep 23;10:15540. doi: 10.1038/s41598-020-72375-y (PMC7511320; doi:10.1038/s41598-020-72375-y)
Supplement: Supplementary file 1 — Supplementary Information. [file 41598_2020_72375_MOESM1_ESM.docx]

**Animated virtual characters to explore audio-visual speech in controlled and naturalistic environments**

Raphaël Thézé, Mehdi Ali Gadiri, Louis Albert, Antoine Provost, Anne-Lise Giraud and Pierre Mégevand

**Supplementary material**

- **Supplementary Movie 1**
- **Supplementary Figure 1**
- **Supplementary Figure 2**

**Supplementary Movie 1.** An example audiovisual speech stimulus (with the sentence “L’objet était dans la base/vase”) is shown with all four possible combinations of phoneme and viseme. In the experiment, participants would be required to indicate by button press whether they heard “base” or “vase” after each trial.

**Supplementary Figure 1.** Visual appearance of the 6 virtual characters designed for the task. There are three men and three women. The voice associated to a character was different between characters and consistent throughout the task.

**Supplementary Figure 2.** Illustration of the various steps in the design process of the characters. First, in A, the physical features of the character are designed with the software Adobe Fuse CC, then, in B, the body is animated in the software Adobe Mixamo. Each character has a pre-defined set of animations, i.e. articulatory movements and facial gesture, which are called with a C# script during the task. That script will animate the character based on the articulatory sequence that was defined for a specific sentence and randomly add the facial micro-gesture.
