# Supplementary figures and images for: Animated virtual characters to explore audio-visual speech in controlled and naturalistic environments
Source: Sci Rep. 2020 Sep 23;10:15540. doi: 10.1038/s41598-020-72375-y (PMC7511320; doi:10.1038/s41598-020-72375-y)

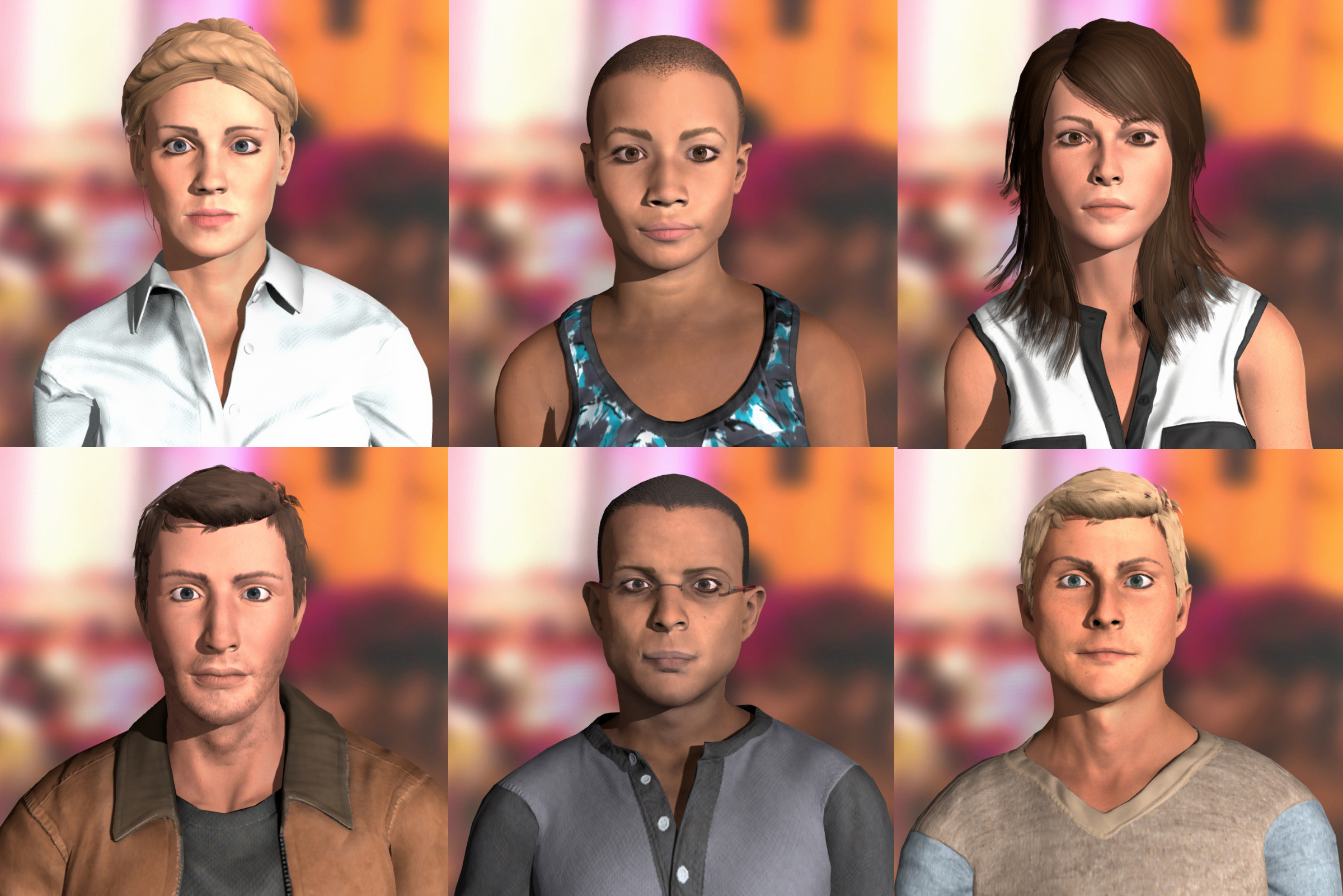

Supplement: Supplementary file 3 — Supplementary Figure 1. [file 41598_2020_72375_MOESM3_ESM.jpg]

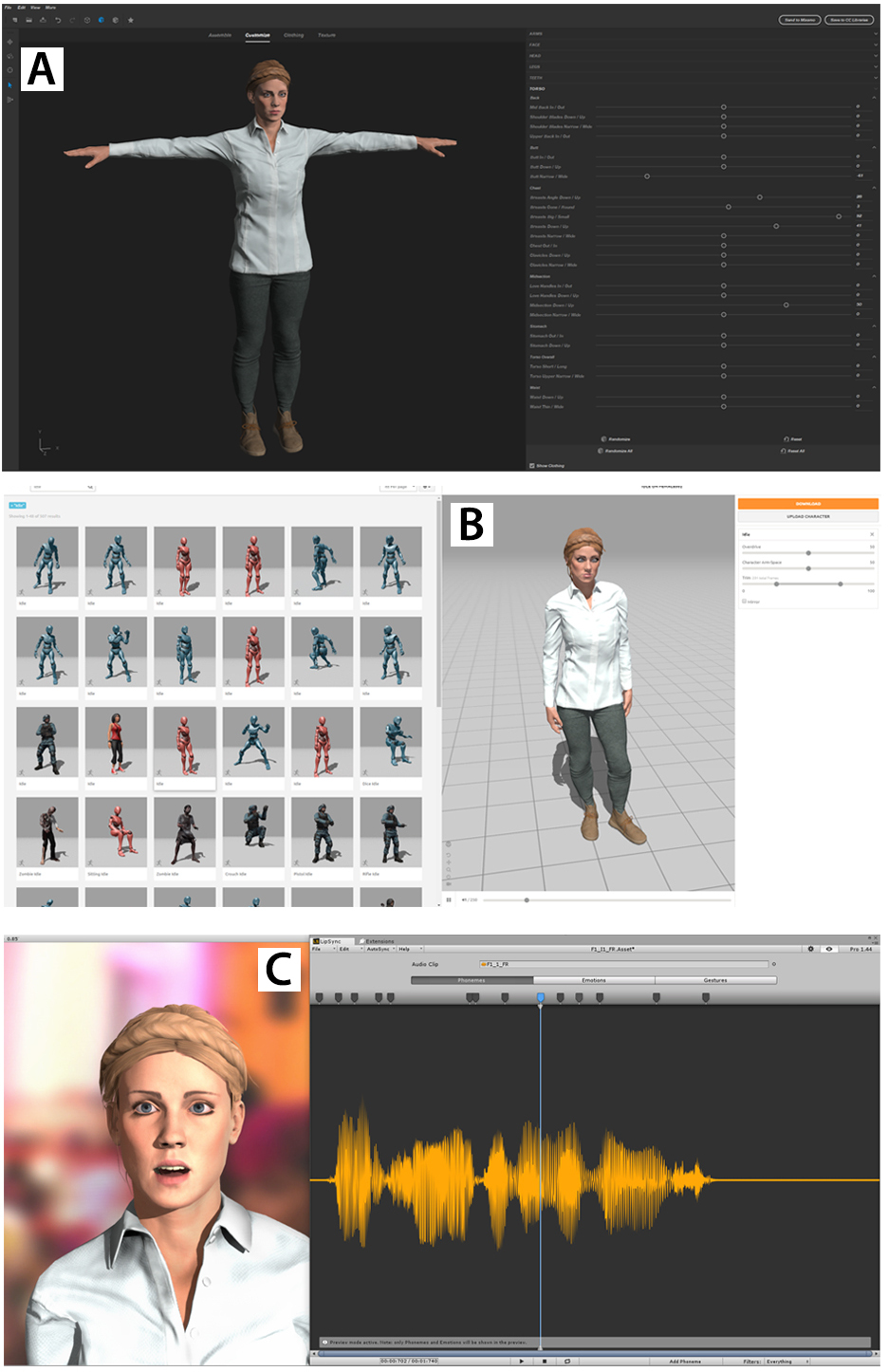

Supplement: Supplementary file 4 — Supplementary Figure 2. [file 41598_2020_72375_MOESM4_ESM.jpg]
